# Siglec Ligands

**DOI:** 10.3390/cells10051260

**Published:** 2021-05-20

**Authors:** Anabel Gonzalez-Gil, Ronald L. Schnaar

**Affiliations:** 1 Department of Pharmacology and Molecular Sciences, Johns Hopkins University School of Medicine, Baltimore, MD 21205, USA; alvarenga@jhmi.edu; 2 Department of Neuroscience, Johns Hopkins University School of Medicine, Baltimore, MD 21205, USA

**Keywords:** immune checkpoint, monocytes, macrophages, NK cells, eosinophils, mast cells, neutrophils, B cells, microglia, myelin associated glycoprotein, sialic acid

## Abstract

A dense and diverse array of glycans on glycoproteins and glycolipids decorate all cell surfaces. In vertebrates, many of these carry sialic acid, in a variety of linkages and glycan contexts, as their outermost sugar moiety. Among their functions, glycans engage complementary glycan binding proteins (lectins) to regulate cell physiology. Among the glycan binding proteins are the Siglecs, sialic acid binding immunoglobulin-like lectins. In humans, there are 14 Siglecs, most of which are expressed on overlapping subsets of immune system cells. Each Siglec engages distinct, endogenous sialylated glycans that initiate signaling programs and regulate cellular responses. Here, we explore the emerging science of Siglec ligands, including endogenous sialoglycoproteins and glycolipids and synthetic sialomimetics. Knowledge in this field promises to reveal new molecular pathways controlling cell physiology and new opportunities for therapeutic intervention.

## 1. Introduction

**Figure 1:**
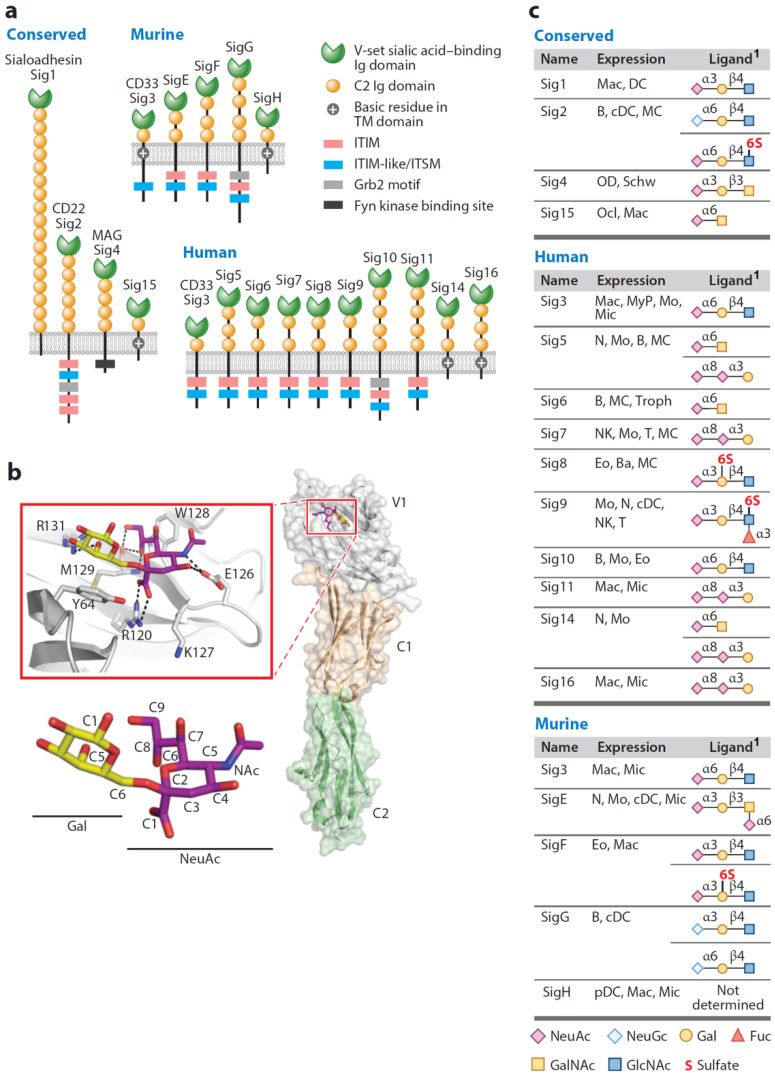
Human and murine Siglecs. (**a**) Structural features of human and murine Siglecs. Each Siglec (Sig) has an N-terminal V-set immunoglobulin (Ig) domain with a conserved sialic acid–binding site, 1-16 additional C2 Ig domains, and a transmembrane domain (TM). Most have intracellular regulatory motifs (ITIM, immunoreceptor tyrosine inhibitory motif; ITSM, immunoreceptor tyrosine switch motif; Grb 2, growth factor receptor-bound 2 motif; Fyn kinase binding site). Some have positively charged amino acids in the transmembrane domain that associate with activating adaptors. (**b**) Crystal structure of a portion of human CD22 with expanded view of the sialic acid–binding site with a bound NeuAcα2–6Gal. The C-1 carboxyl group of the sialic acid binds the conserved arginine (R120). (**c**) Siglec cell type expression and sialoside ligand (B, B cells; Ba, basophils; cDC, conventional dendritic cells; pDC, and plasmacytoid dendritic cells, Eo, eosinophils; Mac, macrophages; MC, mast cells; Mic, microglia; Mo, monocytes; MyP, myeloid progenitor; NK, natural killer cells; N, neutrophils; Ocl, osteoclasts; OD, oligodendrocytes; Schw, Schwann cells; T, T cells; Troph, trophoblasts). Republished with permission of Annual Reviews, Inc., from Duan, S.; Paulson, J.C. Siglecs as Immune Cell Checkpoints in Disease. *Annu. Rev. Immunol.* **2020**, *38*, 365–395 [1]. Permission conveyed through Copyright Clearance Center. ^1^ Glycan ligands shown are exemplary structures known to bind to a given Siglec and are not inclusive. See text for expanded glycan binding specificities.

Siglecs, sialic acid binding immunoglobulin-like lectins, are a family of structurally related animal cell surface glycan binding proteins [1,2,3]. Humans express 14 Siglecs, of which 13 are found on overlapping cell types of the immune system and one (Siglec-4) on myelinating cells in the nervous system (Figure 1, Table 1). Siglecs of the immune system are immune regulatory molecules. Of the 13 immune system Siglecs, 9 carry immunoreceptor tyrosine-based inhibitory (ITIM) motifs that tend to dampen immune responses and 3 have a positive charge in their transmembrane domains that mediates association with DAP12 to activate immune responses. Each Siglec has an outermost (N-terminal) V-set Ig domain containing a sialoglycan binding site centered by a conserved arginine residue that ligates the sialic acid carboxylate (Figure 1a,b) [4]. Different Siglecs bind to distinct sialoglycan ligands to initiate molecular and cellular responses important to the function of the cells on which they are expressed. Knowledge of each Siglec, its particular ligands, and the downstream events triggered by binding provide insights into cell physiology and pathology and potential opportunities for therapeutic intervention [2,5,6] (Table 1).

While all 14 human Siglecs bind sialoglycans [7], the family members diverge in the linkage types and glycan context of the sialic acids to which they bind (examples, Figure 1c). Depending on the Siglec and species, binding may be more or less specific for α2-3, α2-6, and/or α2-8-linked sialic acids, may prefer N-acetylneuraminic acid (Neu5Ac) or N-glycolylneuraminic acid (Neu5Gc), or may require specific underlying sugars in the target glycan with selectivity for the sugar sequence, linkages, and/or specific hydroxyl modifications. Siglecs evolved to distinguish the many ways in which sialic acids are displayed on the larger glycans, of which they are typically the terminal constituents [8]. An additional level of Siglec ligand binding specificity is provided by the glycoproteins or glycolipids on which the recognized glycans are displayed. Precise spacing of multiple Siglec-binding glycan termini on a single glycoprotein or on an apposing cell surface can support multivalent binding by clustered Siglecs to generate remarkably high avidity [9]. Independently, a lectin may bind directly to amino acids on a glycoprotein to complement its glycan binding and enhance binding avidity [10]. Endogenous Siglec ligands are expressed at the appropriate sites of action and with sufficient specificity and affinity to regulate the physiology of the cells on which they are expressed. Knowledge of their structures provides insights into their expression in health and disease. Synthetic Siglec ligands and mimetics, in comparison, can be powerful high-avidity biological and potentially therapeutic tools in probing and regulating Siglec-driven biology [2,4,11]. Both of these classes of Siglec ligands are considered here.

When exploring Siglecs and their ligands, it is important to appreciate their rapid evolution [12]. A subset of Siglecs (Siglec-1, -2, -4 and -15) are evolutionarily conserved between mice and humans. However, most human Siglecs have diverged so significantly from those in rodents that orthologs are unclear. Siglec nomenclature reflects this. Human Siglecs are numbered up to Siglec-16 (Siglec-12 and -13 do not have sialic acid-dependent functions [13]); conserved mouse Siglecs are numbered (Siglec-1, -2, -4, and -15) and non-conserved lettered (Siglec-E, -F, -G, -H). Although some human Siglecs are considered functional paralogs of lettered mouse Siglecs, the functional correlations are inexact, and cross-species conclusions must be thoughtfully evaluated. Siglec-3 (CD33) is named the same in humans and mice but is distinct both in binding specificities and function ([14], see below). An appealing hypothesis for the rapid evolution of Siglecs is that pathogens usurped them, many of which are immune checkpoint inhibitors, to avoid clearance. This would have generated evolutionary pressures that selected for changes in both Siglec ligands and Siglec structures [15,16,17]. For the non-conserved Siglecs, the identity of their endogenous ligands must be considered in a species-specific context.

In this review we explore current knowledge of Siglec ligands, primarily in humans. We first describe some of the experimental approaches used to identify Siglec sialoglycan binding, including glycan arrays, structural elucidation, design and screening of synthetic glycomimetics, cell-based expression systems and extraction, and purification and characterization of endogenous Siglec ligands. These methods have identified basic characteristics of Siglec-binding and revealed high-affinity Siglec-binding sialomimetics. To date, only a handful of endogenous human Siglec ligands have been established. Current knowledge of Siglec ligands for selected human Siglecs are discussed. We anticipate that as exploration of this area expands, increased insight into the distinctive ligands for each Siglec, their physiological and pathological functions, and their therapeutic potential (Table 1) will be forthcoming.

## 2. Experimental Approaches to Identify Siglec Ligands

### 2.1. Siglec-Based Probes

Tagged soluble chimeric Siglec constructs are valuable tools for probing the specificity of Siglec binding. These are typically composed of the N-terminal sialic acid binding V-set domain with or without one or more of the C2 set domains fused with a suitable soluble molecular tag in place of the transmembrane and C-terminal intracellular domains. Nearly all of the mouse and human Siglecs (Figure 1) are commercially available as soluble chimeras with a human Fc tag at the C-terminus replacing the transmembrane and intracellular domains. Fc chimeras have the advantage that they spontaneously form dimers and can be further complexed with commercial fluorescent or enzyme-tagged anti-human-Fc antibodies to increase their multivalency. This is technically important, since the site affinities of Siglecs for monovalent glycans are typically modest (*K*_D_ > 100 µM), with rapid off rates that preclude many types of binding experiments. In nature, Siglecs are expressed multivalently on cell surfaces and bind to multivalent glycans on other cell surfaces or on glycoproteins, resulting in high avidity. Building multivalency into soluble Siglec probes mimics the natural presentation and allows for testing their binding to natural, synthetic, and glycomimetic ligands. A complete set of human Siglec-Fc chimeras engineered to have additional functionality clearly demonstrate this point [18]. By engineering in a Strep-tag sequence, the Fc chimeras were efficiently tetramerized using Strep-Tactin to create octameric multimers that increased binding to cellular targets by nearly 1000-fold compared to the dimeric Fc. In an alternate approach, the extracellular domains of Siglecs were expressed with a C-terminal pentamerizing domain from cartilage oligomeric matrix protein to create high-avidity multivalent probes [19]. The utility of soluble tagged versions of many human and mouse Siglecs is well established.

### 2.2. Glycan Arrays

Microarrays of defined glycans and glycomimetics are valuable tools for evaluating structural requirements for Siglec-sialoglycan binding [20,21,22,23]. Glycan libraries printed on glass slides with up to several hundred defined glycans are simultaneously probed with soluble tagged Siglecs. Siglec binding to glycan microarrays indicates that Siglecs bind sialoglycans with overlapping specificities, some with narrow target binding requirements (e.g., Siglecs-2, -7, -8) and others with what appears to be broader specificity [1]. Published data for Siglec binding to glycan microarrays is referenced in context later in this review. Additional data can be found in a publicly available database with glycan microarray binding of several human and mouse Siglecs [24,25].

While glycan microarray binding is powerful and revealing, broader conclusions must appreciate the limitations of these data. While glycan microarrays have up to several hundred defined glycans, it is estimated that human glycome may contain ~3000 glycoprotein and/or glycolipid binding determinants [26]. If high-affinity binding determinants are missing from the arrays, low affinity binding partners can be misinterpreted as ligands. Likewise, multivalent presentation on most glycan arrays is arbitrarily based on the immobilization technology. The power of entropic forces in specifically arrayed glycans on cell surfaces or glycoproteins may be absent in glycan microarrays [27]. In addition, binding affinity contributions of the carrier (e.g., glycoprotein) backbone are often absent in glycan microarrays. As described in context below, binding to isolated native Siglec ligands can be a million-fold more avid than the site affinity for that Siglec. Nevertheless, glycan microarray binding has proven to be accurate and instructive for many Siglecs.

### 2.3. Sialomimetic Design and Screening

The structures of the primary binding domain (V-set domain) of several human Siglecs have been solved [4]. Structures with bound minimal sialoglycan ligands, each with the sialic acid carboxylate liganded to a conserved arginine residue, provide the basis for designing enhanced sialomimetics, sialosides synthetically modified with unnatural chemical substituents to engage amino acids near the primary sialic acid binding site and increase avidity [28,29]. In silico modeling combined with synthetic chemistry has provided sialomimetics with improved affinity compared to the minimal binding natural sialoglycans. In a complementary approach, sialosides modified at defined positions with libraries of small chemical substituents with various binding properties were screened for Siglec binding to discover high-affinity sialomimetics [22,30,31,32]. An iterative approach combining these methods has identified potent Siglec sialomimetics and revealed the molecular bases for their enhanced avidity [2,4].

### 2.4. Cell-Based Binding Assays

Most Siglecs were initially identified as sialic acid binding proteins based on their ability to bind to erythrocytes in a sialidase-reversible manner [33,34,35,36,37,38,39]. This well-established method to determine sialoglycan recognition and linkage specificity on native glycans [40] was recently updated to explore the structural details of Siglec recognition of sialoglycans in the context of the living mammalian cell surface [41,42,43,44]. Molecular genetic tools were used to stably delete and/or insert glycan biosynthetic genes so as to alter selective classes and linkages of sialoglycans on cultured cells. By quantitatively comparing arrays of genetically altered cells for their ability to bind Siglecs, specific patterns emerged [45]. As an alternate approach, when a cell line with robust Siglec binding was identified, it was subjected to genome-wide gene knockout, isolates with altered Siglec binding were captured, and the disrupted genes identified [43].

### 2.5. Native Siglec Ligand Identification

Identification of native Siglec ligands requires knowledge of endogenous cells and tissues where they are expressed, often followed by physical isolation and identification of the glycans and carriers (proteins, lipids) on which those glycans occur. For human Siglec ligands, this may require human cell or tissue sources in light of their rapid evolution. Examples of Siglec ligands that are retained across mammalian species and those that have diverged will be addressed in context below. Siglec ligands may also vary among cells and tissues from the same species, where different proteins (for example) may carry similar glycan ligands depending on the tissue source. With 14 human Siglecs and potential variations in their ligands depending on cell and tissue types, this is a rich area for discovery that is in its infancy. The area benefits from having robust Siglec-based probes that are useful in determining the tissue and cell distribution of Siglec ligands using Siglec overlay histochemistry. The same tools can be used to identify Siglec ligands in tissue extracts via Siglec overlay of extracted ligands on electrophoretic blots, for example. Affinity isolation using tagged expressed Siglecs linked to beads can be combined with other methods of molecular resolution to isolate native ligands, which can be subjected to proteomic and glycomic analyses.

For Siglec ligands that are retained across species, genetic engineering (e.g., of mice) can be used to probe the phenotypic outcomes of altered Siglec ligand expression in comparison with altered expression of the complementary Siglec. Mutations and polymorphisms in human genes can inform about Siglecs and Siglec ligands even when animal models are unavailable. Examples of both will be described in the Siglec-4 section, below.

In some cases, native Siglec ligands are expressed by human or animal cells in culture. In those cases, genetic modifications have been applied to probe for the genes involved [43]. This may be broadly relevant for cancer cells, which may express sialoglycan ligands to engage and usurp endogenous glycan binding and its downstream effects. Since many Siglecs are immune checkpoint inhibitors, cancer cells expressing Siglec ligands can reduce immune clearance. Knowledge of cancer Siglec structures may provide new avenues to design therapeutics to block them and enhance clearance of the cancer cells. This may be the case whether or not cancer Siglec ligands are related to those on non-diseased tissues.

Enhanced tools for glycan analysis, chemical and chemoenzymatic synthesis, bioinformatics, genetics, and glycan expression are rapidly emerging [46]. Use of these tools to discover Siglec ligands and provide insights into their cell physiology, pathology, and potential therapeutics is accelerating.

## 3. Human Siglec Ligands

### 3.1. Siglec-4 (Myelin Associated Glycoprotein, MAG)

Sequence similarity with two other sialic acid binding proteins, sialoadhesin (Siglec-1) and CD22 (Siglec-2), led to validation of myelin-associated glycoprotein (MAG) as the third immunoglobulin-like sialic acid binding lectin identified [33]. The renaming of the growing Siglec family [7] led to its eventual co-designation as Siglec-4. MAG is expressed exclusively by myelinating cells of the nervous system [47], oligodendrocytes in the central nervous system, and Schwann cells in the peripheral nervous system. These cells enwrap nerve cell axons with multi-layered sheets of myelin membrane, providing insulation and protection. Segmental myelin-wrapped stretches of axon are periodically interrupted by unmyelinated gaps, nodes of Ranvier, where axon ion channels are clustered to support rapid saltatory nerve conduction. Loss of myelin or disruption of molecular organization at nodes of Ranvier leads to impaired nerve conduction, axon degeneration, and accompanying neuropathies [48]. MAG is one of the molecules responsible for maintaining myelin-axon membrane-membrane interactions.

MAG is highly conserved in vertebrates over its entire extracellular domain [49]. Likewise, the major sialoglycans of the vertebrate nervous system, gangliosides, are also highly conserved [50]. Screening MAG for ganglioside binding revealed high specificity for the shared terminal sequence Neu5Acα2-3Galβ1-3GalNAc on major mammalian gangliosides GD1a and GT1b [51]. Subsequent studies using gene-deletion in mice found that mice lacking the N-acetygalactosaminyltransferase (*B4galnt1* gene deletion) responsible for initiating this motif on gangliosides suffered progressive dysmyelination leading to hindlimb paralysis [52]. A direct comparison of *B4galnt1*-null mice with those lacking MAG (*Mag*-null) revealed very similar neuropathies consistent with a loss of axon-myelin connectivity [53]. Congenital disruption of the same gene in humans, *B4GALNT1*, was found to be the cause of one form of hereditary spastic paraplegia [54], a syndrome with symptoms much like those of *B4galnt1*-null mice. Notably, congenital disruption of the *MAG* gene in humans results in a similar syndrome [55]. A very rare human mutation in the single conserved arginine in the MAG V-set domain responsible for sialic acid ligation results in the same neuropathological deficits [56]. These data reveal that, MAG binds to gangliosides GD1a and GT1b to help stabilize myelin-axon interactions.

Studies of the fine specificity of MAG binding to sialoglycans revealed lack of binding to gangliosides bearing the N-glycolyl form of sialic acid (Neu5Gc), or those with a truncated sialic acid glycerol side, altered sialic acid hydroxyl groups, or altered sialic acid carboxylate [57]. Enhanced binding was found when the terminal ganglioside trisaccharide was further substituted with an α2-6-linked sialic acid on the GalNAc, Neu5Acα2-3Galβ1-3(Neu5Acα2-6)GalNAc, the terminus of so-called “α-series” gangliosides such as GQ1bα that are selectively expressed on cholinergic neurons [58]. This same terminus is attached to proteins as the disialyl T antigen, suggesting that in some circumstances O-linked glycoproteins may engage MAG [59]. This is unlikely to be significant for axon-myelin interactions, since selective deletion of the glycolipid-specific N-acetylgalactosaminyltransferase *B4GALNT1* in humans or mice results in the same phenotype as *MAG* disruption.

In addition to stabilizing axon-myelin interactions, MAG binding to axons also limits axon regeneration after traumatic injury in a sialoglycan-dependent manner [60,61]. This led to the search for high-avidity MAG-binding sialomimetics as potential treatments to enhance axon outgrowth after injury [29]. A sialic acid mimetic with sub-micromolar *K*_D_ was synthesized by adding a difluorobenzyl substituent at the 2-position, a fluoroacetate at the 5-position, and a p-chlorobenzamide at the 9-position (Table 2) [62]. The disialyl T tetrasaccharide α-linked to threonine and with a 9-N-fluoro-benzoyl substituent on the α2–6 sialic acid was yet more potent, with a *K*_D_ of 15 nM [63].

### 3.2. Siglec-2 (CD22)

Siglec-2 (CD22) is highly expressed on B cells and is evolutionarily well conserved among mammals [69]. It is an immune inhibitory receptor, with multiple intracellular ITIM motifs [70]. Independent of its sialic acid binding capability, CD22 inhibits B cell responses by associating laterally with the B cell receptor (BCR), down-regulating its activation to protect the organism from overaggressive B cell responses and autoimmunity [71]. Whereas the ITIM motifs of CD22 mediate inhibition of the BCR, its sialoglycan binding capacity often acts, counterintuitively, to reduce those inhibitory effects, as described below. In this case, sialoglycan binding counterbalances ITIM-mediated inhibition [72]. Knowledge of the sialoglycan ligands of CD22 provides a fuller understanding of how sialoglycan binding reverses ITIM inhibition of the BCR.

CD22 (Siglec-2) is highly specific for α2-6-linked sialoglycans [73]. On a glycan array of >50 α2-3- and α2-6-linked sialoglycans, human CD22 bound exclusively to terminal α2-6 sialic acids carried on a variety of glycan scaffolds. Specificity for α2-6-linked sialic acids is due to the structural relationship between the sialic acid carboxylate-ligating arginine on the V-set domain and a tyrosine that stacks against the penultimate galactose (Figure 1b) [74]. Binding of human CD22 to α2-6 linked Neu5Ac and Neu5Gc is equivalent, whereas mouse CD22 is selective for Neu5Gc. Binding of CD22 of both species was enhanced by a nearby sulfate group (Neu5Acα2-6Galβ1-4[6SO3]GlcNAc).

The function of CD22 binding to α2-6-linked sialic acids was confirmed genetically. Mice expressing CD22 with a mutation in the V-set domain arginine required for sialic acid binding had strongly inhibited BCR function [72], as did mice lacking the α2-6 sialyltransferase *St6gal1* [75]. Notably, *St6gal1*-null mice regained BCR function when crossed with mice lacking CD22 [76], indicating that the immunosuppressive outcome of lacking α2-6-linked sialic acids was all CD22 mediated. The conclusion is that α2-6-linked sialoglycan binding by CD22 halts its ability to inhibit the BCR. This becomes especially relevant in potential treatment of autoimmune diseases, as discussed later in this section.

The search for endogenous Siglec-2 (CD22) sialoglycan ligands consistently revealed CD22 itself [77,78,79]. This finding helped explain the above observations. CD22 appears to be in equilibrium between sialic acid dependent homotypic clustering, where it is sequestered from the BCR, and sialic acid independent association with and inhibition of the BCR. Disruption of homotypic (sialic acid dependent) binding releases CD22 to find and inhibit the BCR. Direct evidence for this hypothesis comes from nanoscale dynamic single-particle tracking [80]. Whereas wild type CD22 is organized into characteristic nanoclusters, CD22 molecules lacking sialic acid binding (R120E) were in smaller clusters with higher mobility.

Whereas CD22 itself appears to be the major *cis* ligand on B cells, other sialoglycoproteins also associate with CD22 in a sialic acid-dependent manner [77,78,80,81]. IgM clusters with CD22 at sites of contact between B cells [81]. This association is selective, even though terminal α2-6-linked sialic acids are abundant on many cell surface proteins. Evidently, IgM displays α2-6-linked sialic acids in a particularly high avidity conformation for *trans* recognition by CD22. A second major glycoprotein that associates with CD22 is the heavily glycosylated CD45, an abundant leukocyte-specific cell surface glycoprotein [78,81]. Sialic acid-dependent recruitment of CD22 to the BCR along with BCR antigen enhances CD22-mediated inhibition and even induces tolerance in vivo [79,82].

The ability of CD22-sialoglycan binding to regulate B cell receptor responsiveness provides opportunities for therapeutic design [66,79,83]. Co-delivery of an antigen and a high-affinity CD22 sialomimetic in a liposome resulted in tolerance in mouse models of autoimmune hemophilia and rheumatoid arthritis [82,83]. A synthetic mouse CD22 mimetic with low micromolar affinity, Neu5Gcα2-6Galβ1-4GlcNAc with a 9-N-biphenylacetyl group [64] (Table 2), was synthesized with a lipid aglycone and formulated into liposomes along with antigens related to the target autoimmune diseases, inducing tolerance in vivo. A similar lipid-linked mimetic targeting human CD22, Neu5Acα2-6Galβ1-4GlcNAc carrying a 9-N-*m*-phenoxybenzamide (Table 2) [65], prevented production of targeted antibodies by human memory B cells isolated from rheumatoid arthritis patients [83]. A tri-substituted sialic acid containing a biphenycarboxamide at the 9-position, N-sulfate at the 4-position, and a 3-(propylsulfanyl)propanoic acid glycoside has a remarkable 2 nM affinity for CD22 [66]. When added as a monovalent sialomimetic to human B lymphoma cells stimulated with anti-IgM, the BCR response was increased in a mechanism that has yet to be fully explained. Development of high-affinity sialomimetics for CD22 holds promise for potent Siglec-targeted therapeutics for autoimmune diseases, other immune system disorders, and for targeting immune cell cancers (Table 1) [2,4,84].

### 3.3. Siglec-7

Siglec-7 is an immune inhibitory Siglec that was discovered on natural killer (NK) cells [36], where it has been most extensively studied. Since NK cells are components of the innate immune response to tumors and viruses [85], immune inhibitory Siglec-7 is of interest as a targetable immune checkpoint inhibitor for cancer treatment (Table 1) [86]. Toward this end, Siglec-7 ligands have been identified on cancer cells and Siglec-7 sialomimetics have been developed. Siglec-7 is also expressed on dendritic cells, monocytes, T cells, and mast cells.

Array binding to defined sialoglycans revealed that Siglec-7 preferentially recognizes disialoglycans, with ganglioside GD3 (Neu5Acα2-8Neu5Acα2-3Galβ1-4Glc-) as an example [87,88,89]. Since some cancers overexpress GD3 and related α2-8 linked disialogangliosides, these are potential functional Siglec-7 immune checkpoint inhibitors [87,90]. Structural elucidation of Siglec-7 bound to a related disialoglycan (GT1b) revealed that the terminal α2-8 linked sialic acid carboxylate engaged the conserved arginine on the terminal V-set domain with the penultimate sialic acid engaged by a network of hydrogen bonds [91]. When bound, the glycan turns at the Neu5Acα2-3Gal linkage so that the Galβ1-4Glc residues and aglycon lie along the surface of the binding site stretching toward a hydrophobic patch revealed by a conformational change upon binding. Whether the hydrophobic patch engages the lipid of gangliosides has not been determined.

Branched terminal disialoglycans (e.g., Neu5Acα2-3Galβ1-4(Neu5Acα2-6)GlcNAc/GalNAc) also support Siglec-7 binding on glycan arrays, as do structures with sulfate groups on the Gal and/or HexNAc 6-hydroxyl [87,92]. The occurrence and roles of these glycans in immune cell regulation via Siglec-7 have yet to be established.

Blocking Siglec-7 on NK cells may release them from Siglec-7 mediated inhibition and enhance tumor clearance. Targeting Siglec-7 via multivalent sialoglycans may allow cargo delivery to Siglec-7-bearing cells. To both of these ends, Siglec-7 sialomimetics have been developed [2]. In one powerful screening technology [30], sialoglycans bearing 9-N-alkyne or 5-N-alkyne moieties were arrayed on glass slides and an azide-bearing library of ~100 compounds were covalently attached via click chemistry. The optimal mimetic discovered bore the 5-azidofluorescene click product of the 9-N-alkyne of Neu5Acα2-6Galβ1-4Glc (Table 2). Liposomes bearing the lipid-linked mimetic targeted Siglec-7-bearing cells with high selectivity. A combination of molecular modeling and screening led to a lower molecular weight Siglec-7 sialomimetic, Neu5Ac with a 9-N-ethylsulfonamide, and a clicked biphenyltriazole aglycone, which has low micromolar monovalent affinity (Table 2) [67].

Recent searches for native ligands of Siglec-7 on cancer cells used different approaches that identified the same target sialoglycan [43,93]. Both studies used the well-established NK target line, K562 human chronic myelogenous leukemia cells. In one study [43], K562 cells, which bind Siglec-7 robustly, were subjected to CRISPRi genomic screening to search for genes that, when deleted, resulted in lower Siglec-7 binding. The genomic “hits” included genes involved in sialoglycan biosynthesis and a single glycoprotein, CD43 (leukosialin). In another approach [93], K562 membrane lysates were subjected to affinity capture using Siglec-7-Fc and eluted selectively with dextran modified with pendant Neu5Acα2-8Neu5Ac residues [94]. Proteomic mass spectrometry of the Siglec-7 binding component again revealed CD43. Both studies revealed that modulation of CD43 on K562 cells (and other human leukemia and lymphoma cell lines [43]) altered Siglec-7-mediated NK cell killing. Although the reason why CD43, among all cell surface sialoglycoproteins, is selectively targeted by Siglec-7 remains unresolved, data suggest that clustered groups of sialoglycans may enhance avidity [43]. On other cancer cells, different glycoprotein carriers may carry Siglec-7 binding sialoglycans.

### 3.4. Siglec-8

Siglec-8 is an immunoinhibitory Siglec expressed on human eosinophils, mast cells, and to a lesser extent on basophils [37,95,96]. Clustering of Siglec-8 with antibodies or synthetic glycans induces eosinophil apoptosis and inhibits release of inflammatory mediators from mast cells [97,98,99]. Inhibition is enhanced when the cells are treated with activating cytokines, suggesting that when Siglec-8 is engaged by sialoglycan ligands it functions to resolve ongoing allergic inflammation. This makes Siglec-8 engagement an appealing target for treating allergic diseases (Table 1) [100].

Glycan array binding revealed that Siglec-8 is highly specific, requiring a glycan with both an α2-3-linked sialic acid and a 6-sulfate on the same galactose, Neu5Acα2-3[6SO3]Galβ1-R [101,102,103,104]. Structural studies revealed the molecular basis for this specificity. When the sialic acid carboxylate engages the conserved arginine of the N-terminal V-set domain (R109), the penultimate galactose is positioned via stacking to a tyrosine (Y58) such that its 6-position sulfate makes a salt bridge to another arginine (R56) and hydrogen bonds to a glutamine (Q59) [104]. Binding isotherms revealed that the sulfate increased the site affinity for the monovalent glycan ~30-fold, to ~300 µM.

There is no genetic ortholog of Siglec-8 in rodents or non-primate mammals. However, mouse Siglec-F is expressed on mouse eosinophils and inhibits lung eosinophil accumulation in an experimental allergic challenge model [105]. Although Siglec-F binds to the same sialylated sulfated structures as Siglec-8 on glycan arrays, it binds several other sialoglycans as well [102]. Notably, although Siglec-F binds to ligands on human airways, Siglec-8 does not bind to any ligands on mouse airways [102], suggesting recent evolutionary divergence of both Siglec-8 and its ligands, and narrowing the search for Siglec-8 ligands to human tissues.

A survey of postmortem human lungs and human upper airway surgical samples for Siglec-8 ligands using Siglec-8-Fc overlay histochemistry indicated selective expression on upper airways including bronchus, trachea, and nasal passages [102,106]. Siglec-8 ligands were intensely expressed in submucosal glands, submucosal ducts, and on cartilage, but were notably absent from airway epithelia [102]. Enzymatic treatment revealed that all Siglec-8 ligands on human airways are sialylated keratan sulfate proteoglycans [19]. This is consistent with synthetic ligand binding, since keratan sulfates are chains of repeating monosulfated disaccharides, (Galβ1-4[6SO3]GlcNAc)_n_, that can be terminated with α2-3-linked sialic acid and can carry additional 6-linked sulfates on the Gal residues [107]. Two different protein carriers were found to carry these sialylated keratan sulfate Siglec-8 ligands on cartilage and in submucosal glands [19,108]. Affinity purification of Siglec-8 ligand from postmortem human trachea identified the major cartilage protein aggrecan [19]. Although aggrecan consistently carries keratan sulfate chains, only a portion of aggrecan is post-translationally modified to carry Siglec-8 ligands, designated here as aggrecan^S8L^. Treatment of eosinophils with affinity-purified aggrecan^S8L^ revealed increased apoptosis compared to control aggrecan without the intact Siglec-8 ligand.

In contrast to aggrecan in cartilage, a soluble proteoglycan was discovered to carry Siglec-8 ligand in submucosal glands and, via submucosal ducts, onto the airway surface [108]. Although postmortem airways and surgical samples were washed free of the soluble mucus Siglec-8 ligand produced in submucosal glands and secreted as a soluble sialoglycan onto the airway mucus layer was abundant in saline washes of human nasal airways, a standard procedure performed prior to sinus surgery. Affinity purification of Siglec-8 ligand from human airway secretions led to identification of DMBT1 (deleted in malignant brain tumor 1) as the ligand carrier. DMBT1 is an abundant secreted glycoprotein also known as a salivary scavenger and agglutinin (SALSA or SAG) or gp340 [109]. A portion of this abundant submucosal gland-secreted protein is specifically glycosylated with Siglec-8 ligands (DMBT1^S8L^). Affinity-purified DMBT1^S8L^ had picomolar affinity for Siglec-8 [108], a million-fold higher than monovalent glycan, suggesting that the sialylated keratan sulfate chains on DMBT1^S8L^ responsible for binding are displayed in multivalent groupings or in association with other components that greatly enhance Siglec-8 binding. Elevated expression of DMBT1^S8L^ was observed in nasal tissue secretions from patients with eosinophilic inflammation [106,110], suggesting an active cellular response to resolve ongoing eosinophilic inflammation.

To develop high affinity Siglec-8 selective mimetics, a glycan microarray library of synthetic 9-N-sulfonyl sialoside analogues was screened, with the best ligand being 9-N-(2-naphthyl-sulfonyl)-Neu5Acα2-3[6SO3]Galβ1-4GlcNAc (Table 2), incorporating the best sialic acid mimetic with the minimum native sialoglycan scaffold [31]. Mouse Siglec-F also bound with highest avidity to the same structure. The optimal ligand bound only to Siglec-8 and Siglec-F, with no binding to other human or mouse Siglecs. The same 9-N-(2-naphthyl-sulfonyl) sialic acid synthesized as a glycoside with a 3-methylsulfate-cyclohexanol aglycone (Table 2) provided a monovalent *K*_D_ of 15 µM [68].

### 3.5. Siglec-9

Siglec-9 is an immunoinhibitory Siglec broadly expressed on human leukocytes including monocytes, macrophages, neutrophils, dendritic cells, and subsets of NK cells, B cells, and T cells [4,111]. Its immune inhibitory and checkpoint functions have been studied in relation to inflammation and cancer immune evasion (Table 1) [112,113]. Clustering of Siglec-9 on the cell surface of neutrophils with a monoclonal antibody or polyvalent sialoglycan ligand induces neutrophil death, an effect that is enhanced when neutrophils are activated [114,115], and Siglec-9 engagement on macrophages inhibits phagocytosis [112].

Siglec-9 glycan specificity for synthetic sialosides is relatively broad with α2-3 and α2-6 sialoglycans supporting binding [88,111]. On glycan arrays, selective binding was seen to sialyl Lewis x (SLe^x^, Neu5Acα2-3Galβ1-4[Fucα1-3]GlcNAc) and 6-sulfo-SLe^x^ (Neu5Acα2-3Galβ1-4[Fucα1-3][6SO3]GlcNAc) [102]. Siglec-9 also bound to sialylated glycolipids (gangliosides) having terminal α2-3-linked sialic acids [102], and to the glycosaminoglycan hyaluronic acid (GlcNAcβ1-4GlcAβ1-3)_n_ [116], which lacks sialic acid.

A search for native Siglec-9 ligands that modulate inflammation led to the major erythrocyte sialoglycoprotein glycophorin, which inhibited neutrophil activation [117]. In sickle cell disease, this inhibitory effect is reduced and neutrophils are more prone to activation, contributing to pathophysiology [118]. Siglec-9-mediated neutrophil inhibition was also observed when neutrophils were incubated with high molecular weight hyaluronic acid, which is expressed abundantly in humans and by certain human pathogens [116]. Several mucins have also been identified as carriers of Siglec-9 ligands. In human upper airway tissues (and a human airway cell line), Siglec-9 bound to glycans on the major airway mucin MUC5B [106]. Expression of this Siglec-9 ligand was upregulated in human inflammatory airway disease and in human airway cells in vitro after exposure to bacterial lipopolysaccharide (LPS), a pattern recognition receptor ligand. Siglec-9 ligands have also been found in human aorta and to be upregulated in human vascular endothelial cells grown in high glucose [119]. In cancer, hyper-sialylation and hyper-glycosylation on mucins is common [120]. MUC1 and MUC16 are post-translationally modified in the tumor environment to carry immune inhibitory Siglec-9 ligands [121,122,123], making Siglec-9 ligands inviting targets to enhance immune surveillance in cancer.

Therapeutic opportunities for both engaging and inhibiting Siglec-9 are under investigation. Engaging Siglec-9 (e.g., by multivalent ligands) may dampen immune responses in inflammatory diseases, whereas blocking Siglec-9 binding may release immune inhibition and enhance cancer immune surveillance. A selective Siglec-9 binding sialomimetic with a diphenymethyl triazole covalently attached at the C5 of sialic acid of Neu5Ac9α2-6Galβ1-4Glc (Table 2) was synthesized and selected by printed sialomimetic library screening [22]. This Siglec-9 sialomimetic was clustered on a synthetic polypeptide with a lipid tail to generate a polyvalent cell surface-deliverable Siglec-9-targeting artificial glycopeptide. When delivered to the surface of intact cells in vitro, the mimetic inhibited macrophage phagocytosis, neutrophil extracellular trap formation, and induced neutrophil apoptosis [112,115].

### 3.6. Siglec-1 (Sialoadhesin, CD169)

Siglec-1 is expressed predominantly on macrophages and is conserved among mammals [1,124,125]. It is involved in uptake of sialylated pathogens (including HIV-1), antigen presentation, and regulating immune tolerance to self-antigens. Siglec-1 does not contain a conserved intracellular signaling motif and is internalized by clathrin-dynamin-dependent endocytosis. With 17 Ig-like domains, Siglec-1 is the longest Siglec, hypothesized to extend away from the cell surface to avoid *cis* interactions and support *trans* interactions with sialoglycans outside of the cell [125]. Endogenous ligands for Siglec-1 have been identified on T-cells and dendritic cells as well as pathogens, and liposomal nanoparticles containing Siglec-1-targeting sialomimetics have been developed as tools for antigen delivery.

Binding to synthetic glycans or linkage-specific re-sialylated erythrocytes revealed that Siglec-1 bound to a variety of sialoglycans, but with a preference for α2-3 linked Neu5Ac [33,88,126,127]. Linkage selectively was further confirmed when Siglec-1 ligands on primary murine T cells were nearly completely lost upon treatment with α2-3-specific sialidase [128]. The crystal structure of Siglec-1 V-set domain bound to Neu5Acα2-3Galβ1-4Glc revealed that most of the direct protein contacts were with the sialic acid, including by the conserved V-set arginine (R97) to the sialic acid carboxylate and five surrounding amino acids that ligated different atoms on the sialic acid directly to generate hydrogen bonds and hydrophobic interactions [129].

Among native Siglec-1 ligands, affinity capture from a murine T lymphoma cell line identified the heavily glycosylated proteins CD43 and PSGL-1 [130]. However, expression of Siglec-1 ligands on primary mouse T cells was independent of either [128], perhaps reflecting the propensity of cancer cells to overexpress sialoglycans that can engage Siglecs. In this light, Siglec-1 ligand was affinity captured from human breast cancer cell lines and identified as a glycoform of the common cancer-associated epithelial mucin MUC1 [131]. As for other Siglecs, expression of Siglec-1 ligands with the preferred structure and presentation may occur on different protein carriers.

Siglec-1 ligands, natural and synthetic, have been explored as a way to target antigens to macrophages, which are antigen-presenting cells. Liposomes carrying a peptide antigen and the native Siglec-1 ligand GM3 (Neu5Acα2-3Galβ1-4Glc-ceramide) targeted Siglec-1-expressing macrophages in vivo, resulting in a more robust immune response [132]. Siglec-1 sialomimetic ligands with higher affinity and specificity were developed via sialoglycan mimetic array screening and in silico-aided design [28,133]. On the scaffold of Neu5Acα2-3Galβ1-4GlcNAc, addition of a 9-N-(4H-thieno [3,2-c]chromene-2-carbonyl) group to the sialic acid C9 (Table 2) enhanced binding affinity to sub-micromolar IC_50_, with selectivity for Siglec-1 over other human or mouse Siglecs [28]. Liposomes carrying a lipid-linked form of this mimetic effectively targeted macrophages in vivo for delivery of antigen and immune modulators [134,135].

### 3.7. Siglec-3 (CD33)

Human Siglec-3 (hSiglec-3) is an immunoinhibitory receptor expressed on myeloid progenitor cells, monocytes, macrophages, mast cells, dendritic cells, and brain microglia [2]. It is also highly expressed in acute myeloid leukemia (AML), leading to the use of anti-CD33 antibody carrying a cytotoxic drug for cancer treatment [136,137]. Although CD33 is found in mice (mSiglec-3), it is not included among the conserved Siglecs [1,3,138] since *SIGLEC3* and *Siglec3* gene do not show the expected phylogenetic relationship [12], the proteins diverge in sialoglycan binding [139], and the proteins have different transmembrane and intracellular signaling domains [140,141] leading to functional divergence [14].

Binding to synthetic glycans or linkage-specific re-sialylated erythrocytes revealed that hSiglec-3 bound to both α2-3-linked and α2-6-linked sialoglycans [34,88,126]. Binding to surface-adsorbed gangliosides revealed that hSiglec-3 bound to biantennary sialic acid termini on GD1a and GT1b and yet better to the triantennary sialic termini of GQ1bα. Use of a more extensive glass slide sialoglycan array revealed robust and highly specific binding to the sialylated sulfated sequence Neu5Acα2-3[6SO3]Galβ1-4GlcNAc, the same glycan that bound Siglec-8 [45]. In support of the latter structure as a potential cell surface ligand, binding of hSiglec-3 to human HEK293 cells was absent unless they were transfected with the galactose 6-O-sulfotransferase gene *CHST1* [45]. Deletion of *ST3GAL4* in *CHST1*-transfected HEK293 cells reduced binding, implicating a sialylated galactose-6-sulfated structure.

To target Siglec-3 in vivo, a highly selective and high affinity ligand was synthesized using iterative sialomimetic screening [65]. The highest affinity structure was based on a Neu5Acα2-6Galβ1-4Glc scaffold with the sialic acid di-substituted with a 4-cyclohexyl triazole at the C5 position and a 9-N-4-hydroxy-3,5-mimethylbenzoic acid amide at the C9 (Table 2). The resulting sialomimetic had relatively high site affinity (~100 µM [18,142]) and was hSiglec-3 specific. In an example of its therapeutic potential, the hSiglec-3-targeted sialomimetic was covalently attached to a lipid and incorporated into liposomes alongside a mast cell antigen. When delivered together in the same liposome, the antigen bound to sensitized mast cells while the sialomimetic recruited inhibitory hSiglec-3 to the site, inhibited mast cell degranulation, and blocked anaphylaxis [143]. The preclinical model in this study was a mouse line expressing hSiglec-3 on mast cells, a model of humanized Siglec mice that is gaining utility for other Siglecs in preclinical studies [144,145].

### 3.8. Siglec-11

Siglec-11 is an inhibitory Siglec shared among primates (but not rodents) that is expressed on macrophages and, exclusively in humans, on brain microglia [146,147,148]. Binding to defined sialoglycans revealed robust binding to oligo- and polysialic acids (Neu5Acα2-8Neu5Acα2-8)_n_ [146,148,149,150]. In humans, different Siglec-11 splice variants are expressed on macrophages (5 Ig-like domains) and microglia (4 Ig-like domains), with the microglial form displaying enhanced polysialic acid binding [150]. Polysialic acid is expressed on a narrow group of proteins, with neural cell adhesion molecule (NCAM) being the major polysialic carrier in the brain [50]. Engagement of Siglec-11 with polysialic acid inhibits inflammatory responses of both macrophages and microglia [148,149], and Siglec-11 binds specifically to polysialic acid on the surface of nerve cells [148,150]. Together, these data support the conclusion that polysialic acid on NCAM (or other protein carriers) is an endogenous ligand for Siglec-11.

## 4. Conclusions

Each Siglec in the context of the cells on which it is expressed binds to sialoglycan ligands in its extracellular milieu to drive cell physiology and pathology. The search for Siglec-mediated cell and tissue responses and the sialoglycans that drive these regulatory molecules is a relatively new area of investigation but has already generated the promise of novel therapeutic targets and approaches (Table 1) [1,2,5,11,86,96,100,151]. The recent expansion of knowledge and technology in the glycosciences promises to provide expanding avenues for exploration, discovery, and applications.

## Figures and Tables

**Table 1:** Disease targets of Siglec and anti-Siglec therapies ^1^.

Siglecs	Expression	Diseases
Siglec-1/ sialoadhesin/CD169	Macrophages	Cancers Autoimmunity Infectious disease
Siglec-2/CD22	B cells	B-cell lymphoma systemic lupus erythematosus, sepsis Rheumatoid arthritis Type 1 diabetes
Siglec-3/CD33	Myeloid progenitors Monocytes Macrophages Mast cells Microglia Dendritic cells	Acute and chronic myeloid leukemias Myelodysplastic syndromes Alzheimer’s disease Mast cell-dependent anaphylaxis Systemic mastocytosis
Siglec-4/MAG	Oligodendrocytes Schwann cells	Traumatic nerve injury Neuropathy
Siglec-5	Monocytes Neutrophils Macrophages Dendritic cells	Neutrophil disorders
Siglec-6	Placenta (trophoblasts) B cells	Preeclampsia
Siglec-7	NK cells Monocytes Macrophages Mast cells Dendritic cells	Systemic mastocytosis Mast cell leukemia cancers
Siglec-8	Eosinophils Mast cells Basophils	Allergic asthma Eosinophilic gastritis Chronic urticaria
Siglec-9	Monocytes Macrophages Neutrophils Dendritic cells NK cells (subset) T cells (subsets)	Cancers chronic obstructive pulmonary disease Asthma Rheumatoid arthritis
Siglec-10	B cells Macrophages	Sepsis Cancers Graft-vs-host Disease
Siglec-11	Microglia Macrophages	Neurological disorders
Siglec-14	Neutrophils Monocytes Macrophages	systemic lupus erythematosus chronic obstructive pulmonary disease
Siglec-15	Osteoclasts Macrophages	Cancers Osteoporosis
Siglec-16	Macrophages	

^1^ Reprinted in modified form from Murugesan, G.; Weigle, B.; Crocker, P.R. Siglec and anti-Siglec therapies. *Curr. Opin. Chem. Biol.* **2021**, *62*, 34–42 [2] with permission from Elsevier.

**Table 2:** Endogenous Siglec ligands and Siglec-targeting sialomimetics.

Siglec	Endogenous Ligands ^1^	Sialomimetics	[References]
Siglec-1	CD43^S1L^, PSGL-1^S1L^, MUC1^S1L^	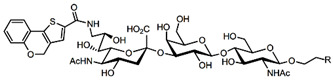	[28]
Siglec-2 (CD22)	CD22, IgM^S2L^, CD45^S2L^	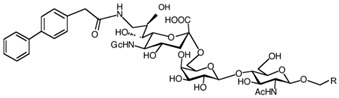	[64]
		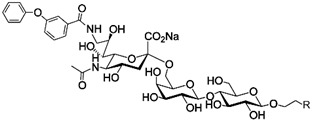	[65]
		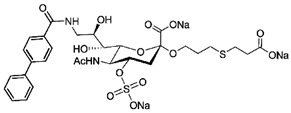	[66]
Siglec-3 (CD33)		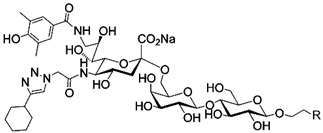	[65]
Siglec-4 (MAG)	GD1a, GT1b, GQ1bα	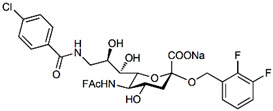	[32]
		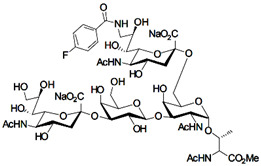	[63]
Siglec-7	CD43^S7L^, GD3	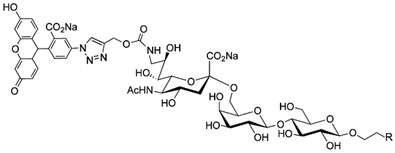	[30]
		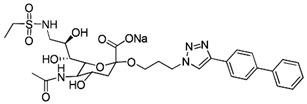	[67]
Siglec-8	aggrecan^S8L^, DMBT1^S8L^	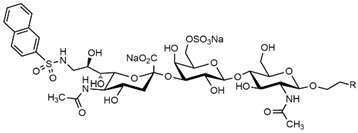	[31]
		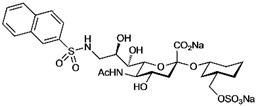	[68]
Siglec-9	glycophorin^S9L^, hyaluronic acid, MUC5B^S9L^, MUC1^S9L^, MUC16^S9L^	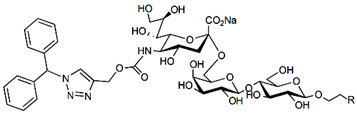	[22]
Siglec-11	polysialic acid (NCAM)		

^1^ Each glycoprotein identified as a Siglec ligand exists as multiple glycoforms, only a subpopulation of which carry the glycans required to bind that Siglec. To designate the Siglec-binding glycoform(s), the superscript S#L is added.

## Data Availability

Not applicable.
